# Role of Hepatic-Specific Transcription Factors and Polycomb Repressive Complex 2 during Induction of Fibroblasts to Hepatic Fate

**DOI:** 10.1371/journal.pone.0167081

**Published:** 2016-11-30

**Authors:** Shima Rastegar-Pouyani, Niusha Khazaei, Ping Wee, Abdulshakour Mohammadnia, Moein Yaqubi

**Affiliations:** 1 Institute of Medical Biotechnology, National Institute of Genetic Engineering and Biotechnology (NIGEB), Tehran, Iran; 2 Department of Medical Genetics and Signal Transduction Research Group, Faculty of Medicine and Dentistry, University of Alberta, Edmonton, AB, Canada; 3 Department of Human Genetics, Division of Hematology and Oncology, Faculty of Medicine, McGill University, Montreal, Quebec, Canada; 4 Ludmer Centre for Neuroinformatics and Mental Health, McGill University, Montreal, Quebec, Canada; 5 Douglas Mental Health University Institute, McGill University, Montreal, Quebec, Canada; University of Texas at Austin Dell Medical School, UNITED STATES

## Abstract

Direct reprogramming using defined sets of transcription factors (TFs) is a recent strategy for generating induced hepatocytes (iHeps) from fibroblasts for use in regenerative medicine and drug development. Comprehensive studies detailing the regulatory role of TFs during this reprogramming process could help increase its efficiency. This study aimed to find the TFs with the greatest influences on the generation of iHeps from fibroblasts, and to further understand their roles in the regulation of the gene expression program. Here, we used systems biology approaches to analyze high quality expression data sets in combination with TF-binding sites data and protein-protein interactions data during the direct reprogramming of fibroblasts to iHeps. Our results revealed two main patterns for differentially expressed genes (DEGs): up-regulated genes were categorized as hepatic-specific pattern, and down-regulated genes were categorized as mesoderm- and fibroblast-specific pattern. Interestingly, hepatic-specific genes co-expressed and were regulated by hepatic-specific TFs, specifically *Hnf4a* and *Foxa2*. Conversely, the mesoderm- and fibroblast-specific pattern was mainly silenced by polycomb repressive complex 2 (PRC2) members, including *Suz12*, *Mtf2*, *Ezh2*, and *Jarid2*. Independent analysis of both the gene and core regulatory network of DE-TFs showed significant roles for *Hnf4a*, *Foxa2*, and PRC2 members in the regulation of the gene expression program and in biological processes during the direct conversion process. Altogether, using systems biology approaches, we clarified the role of *Hnf4a* and *Foxa2* as hepatic-specific TFs, and for the first time, introduced the PRC2 complex as the main regulator that favors the direct reprogramming process in cooperation with hepatic-specific factors.

## Introduction

Currently, liver transplantation is the only approved strategy for the treatment of patients suffering from liver failure. Every year, the number of required livers for transplantation exceed the number of donors [1,2]. The limit in the number of liver donors, high cost, and rejection of transplanted tissue require alternative strategies for tackling liver diseases [1]. Currently, cellular differentiation and direct reprogramming are two alternative strategies for the generation of hepatocytes. The differentiation of embryonic stem cells (ESCs) and induced pluripotent stem cells (iPSCs) is a multi-stage process that provides an unlimited number of hepatocytes, but poses risks for tumorigenesis, due to the possibility of residual undifferentiated cells [1,3,4]. On the other hand, the direct induction of somatic cells to a hepatic fate is a single step strategy for the generation of mature cells without tumorigenesis risks. Direct reprogramming is also faster and safer in comparison with the differentiation of pluripotent stem cells (PSCs) into hepatocyte-like cells [5,6].

Collectively, two approaches have been used to directly reprogram somatic cell identity. The first approach uses Yamanaka factors, Oct4/Pou5f1, Sox2, Myc, and Klf4, beside additional factors that support direct conversion. The second approach involves applying a list of lineage specific transcription factors (TFs) and systematically eliminating unnecessary factors in order to achieve the most efficient combination. Several groups have used these approaches to generate different cell types, including neuronal cells [7–13], neural stem cells [14–21], cardiomyocytes [22–25], and hepatocytes [5,6,26–29]. In relation to the liver, Sekiya & Suzuki (2011) have used the lineage-specific factor Hnf4a in combination with one of Foxa1, Foxa2, or Foxa3 to generate functional induced hepatocytes (iHeps) [6]. Lim et al (2016) used a combination of Klf4 and Myc as their Yamanaka TFs along with Hnf4a as their hepatic-specific factor to drive the direct conversion of fibroblasts into iHeps. This group was also able to obtain iHeps from fibroblasts by combining small molecules with Hnf4a [28]. Despite the extensive use of TFs for the generation of hepatocyte-like cells through direct reprogramming, the molecular mechanisms by which different TFs regulate the gene expression program have not been well understood.

The recent trend of generating large amounts of high quality expression data sets through high throughput technologies allows the unique opportunity to dissect the molecular mechanisms of gene regulation by analyzing the data using systems biology and bioinformatics approaches. Computational approaches are applicable to find the main regulators of gene expression and to dissect their behavior in different cellular transitions, including during differentiation, reprogramming, and direct reprogramming [30–33]. Previously, several strategies have been suggested to predict the TFs involved in developmental processes. For example, CellNet has been used to compare the expression profile of *in vivo* and *in vitro* counterparts of the same cell, in order to predict TFs that can potentially increase the efficiency of differentiation [34]. Also, D’Alessio et al. (2015) has proposed a systematic approach for finding cell identity regulators for a wide range of cells through the comparison of gene expression profiles [35]. Another current strategy is through the use of Mogrify, which introduced a computational framework for predicting TFs for 173 cell types and 134 tissues [36]. In our approach, besides predicting the TFs involved during differentiation, reprogramming, and direct reprogramming, we are also able to analyze their mechanisms during the regulation of the gene expression program and in their biological processes, and to rank their significance based on different computational factors [30,31,37]. In spite of the availability of such powerful strategies for predicting and dissecting the role of TFs during developmental processes as well as the availability of experimentally-obtained highly qualified expression data sets from the induction of fibroblast cells to cells of a hepatic fate, there has not been a comprehensive study performed on the regulation of gene expression during the direct conversion of fibroblasts into iHeps.

In this study, we established and investigated the gene regulatory network (GRN) underlying the induction of hepatic fate from fibroblasts. Collectively, we used eight independent comparisons from six high quality expression data sets to understand the role of TFs in regulating the gene expression program and in biological processes duing the generation of iHeps from fibroblasts. Our results show two distinct patterns in the regulation of gene expression program: up-regulated genes were categorized as hepatic-specific genes and down-regulated genes were categorized as mesoderm- and fibroblast-specific genes. Interestingly, the hepatic-specific gene expression pattern was co-expressed and regulated by hepatic-lineage TFs, especially *Hnf4a* and *Foxa2*. Furthermore, the mesoderm and fibroblast gene expression pattern were mainly regulated by PRC2 members, including *Suz12*, *Mtf2*, *Ezh2*, and *Jarid2*. In summary, for the first time, we clarify the role of hepatic-lineage factors in the regulation of the gene expression program and introduce the PRC2 complex as a significant player during the direct conversion of fibroblasts into iHeps. We believe that our results improve our understanding on the regulation of the gene expression program that occurs duing the generation of iHeps from fibroblasts, and that these results will be useful for achieving a higher efficiency of this direct reprogramming.

## Material and Methods

**Fig 1:**
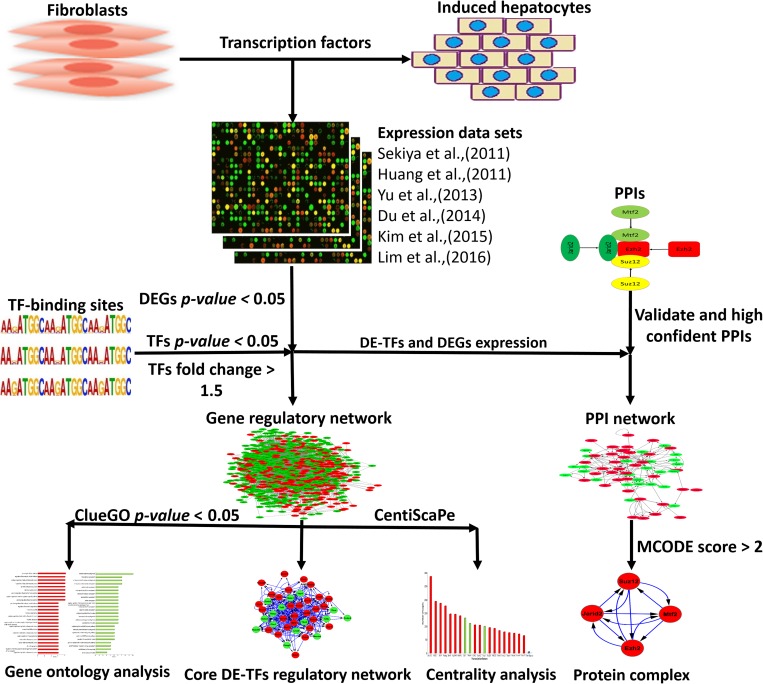
Schematic diagram of analysis expression data sets for direct conversion of fibroblasts into iHeps. Common differentially expressed genes (DEGs) were identified across different data sets. The list of common DEGs were used to identify differentially expressed TFs (DE-TFs) and construct gene regulatory and protein-protein interaction (PPI) networks. The constructed networks were subjected to different analyses, for example, centrality and ontology analyses were conducted on the gene regulatory network.

After careful literature mining for available expression data sets regarding the generation of hepatocyte-like cells from fibroblast, we chose six independent data sets. Data sets with accession numbers GSE29725, GSE23635, GSE67362, GSE52566, GSE48486, and GSE54066 were downloaded from Gene Expression Omnibus (GEO) for analysis of gene expression regulation during induction of hepatic fate from fibroblasts [5,6,26–29](Table 1, Fig 1). For each data set, comparisons between samples were defined and then normalized data were loaded into Flexarray software in order to detect differentially expressed genes (DEGs) and their GEO platforms were downloaded for annotations [38]. To find DEGs, the fold change algorithm was applied for each data set and *p-values* were computed for the list of all genes in each data set, with *p-value* <0.05 set as the filter. Finally, the lists of DEGs from all data sets were combined to identify common DEGs across all datasets. An additional criterion for considering a gene a DEG was that the gene needed to be present in at least four independent data sets. The human microarray data set with accession number GSE54066 was analyzed separately and was used to compare between human and mouse organisms. For transcription factors (TFs), because low level changes in their expressions can lead to dramatic alterations in the gene expression program, we set different criteria for TFs to be considered differentially expressed TFs (DE-TFs). We set a value of 1.5 fold changes or more for TFs to be considered DE-TFs. Furthermore, DE-TFs should also show the same pattern in at least three independent comparisons from the merged data sets.

**Table 1:** Expression data sets used for mouse and human organisms and their experimental design.

Experiment	Organism	Comparison	Accession number	Technology
**Sekiya et al., (2011)**PMID:21716291	Mouse	Three samples of iHeps (Retrovirus vector expressing *Hnf4a* in combination with one of *Foxa1*, *Foxa2* and *Foxa3* factors) vs. three samples of MEFs	GSE29725	Agilent-014868 Whole Mouse Genome Microarray 4x44K G4122F
**Huang et al., (2011)**PMID:21562492	Mouse	Four samples of iHep (from p19arf-null TTF1) (Lentivirus vector expressing *Gata4*, *Hnf1a* and *Foxa3*) vs. three samples of p19arf-null TTF	GSE23635	Agilent-014868 Whole Mouse Genome Microarray 4x44K G4122F
**Kim et al., (2015)**PMID:26503743	Mouse	Two samples of iHeps (Episomal vector expressing *Gata4*, *Hnf1a* and *Foxa3*) vs. MEF	GSE67362	Affymetrix HT MG-430 PM Array Plate
**Kim et al., (2015)**PMID:26503743	Mouse	Two samples of iHeps (Viral vector expressing *Gata4*, *Hnf1a* and *Foxa3*) vs. MEF	GSE67362	Affymetrix HT MG-430 PM Array Plate
**Lim et al., (2016)**PMID:27149847	Mouse	Two samples of Hnf1a transduced (Retrovirus vector expressing *Hnf4a* and *Foxa3*) + small molecules MEFs vs. two samples of MEFs	GSE52566	Affymetrix HT MG-430 PM Array Plate
**Lim et al., (2016)**PMID:27149847	Mouse	Three samples of four factor iHeps (Retrovirus vector expressing *Hnf4a*, *Foxa3*, *Myc* and *Klf4*) vs. two samples of MEFs	GSE52566	Affymetrix HT MG-430 PM Array Plate
**Yu et al., (2013)**PMID: 23871605	Mouse	Four samples of iHep stem cells (from p19arf-null TTF1) (Lentivirus vectorexpressing *Hnf1a* and *Foxa3*) Vs. two samples of MEFs	GSE48486	Agilent-014868 Whole Mouse Genome Microarray 4x44K G4122F
**Du et al., (2014)**PMID: 24582926	Human	Three samples of iHeps (lentivirus vector expressing *Hnf1a*, *Hnf4a*, *Hnf6*, *Atf5*, *Prox1* and *Cebpa*) vs. Human Embryonic Fibroblast	GSE54066	Illumina HiSeq 2000

### Clustering analysis of DEGs

To determine the co-expression pattern and expression relation of DEGs during the induction to a hepatic cell fate from fibroblasts, we used Gene Cluster software and visualized our results using Java TreeView [39,40]. We used hierarchical clustering, which is a strong method for analyzing high throughput expression data. Similarity metrics were calculated for both genes and arrays. To measure the similarity of both arrays and genes, correlation (un-centered) was used.

### Construction of gene regulatory and TF protein-protein interaction networks

There are freely available databases which contain the data of analyzed transcription factor binding sites. This data can be used to identify DEG regulators. For example, ChEA deposits the data of binding sites for 234 TFs obtained from 274 publications, which taken together contains 564,752 regulatory interactions for TFs [41]. This database is mainly composed of TF binding sites obtained from ChIP-chip, ChIP-seq, ChIP-PET, and DamID techniques. In this study, we submitted our list of DEGs to the ChEA database and filtered results based on the *p-value*, keeping the genes with *p-value* <0.05 (Fig 1). Furthermore, another level of filtering was done by applying expression data on genes to select those genes that have expression fold change of 1.5. The TFs meeting these criteria were then considered to be differentially expressed TFs (DE-TFs). To provide a more accurate, informative, and comprehensive analysis, apart from regulatory interactions which exist between protein and DNA, we needed the interactions which exist between TFs themselves in order to construct TF protein-protein interaction networks. In this regard, we used the BioGRID database, which contains experimentally validated protein-protein interactions data (796,767 interactions from more than 50,000 published studies) [42], and also a STRING database which bears both validated and predicted protein-protein interactions [43] (Fig 1). To increase the confidence of obtained interactions from the STRING database, we set a confidence score of 0.7 (high confidence). For both of these databases, downloaded interactions were filtered with the expression data to find and use only meaningful interactions. Protein-protein interactions were loaded into Cytoscape software to construct and visualize the protein-protein interaction networks.

To construct the gene regulatory network (GRN) and the protein-protein interaction network for the direct reprogramming of fibroblasts into iHeps, we integrated TF binding sites, protein-protein interactions, and the gene expression data into the Cytoscape software.

### Ontology analysis of DEGs and constructed networks

To assign the function of DEGs and the constructed GRN to defined categories in the form of the most affected biological function, we used we used the ClueGO and CluePedia Apps [44,45] for the Cytoscape software [46]. In the advanced statistical option of the tools, Two-sided hypergeometric test was selected to calculate the importance of each term and Bonferroni step-down was used for *p-value* correction. Significant terms can be defined as those terms that have *p-value* <0.05 (Fig 1) [44,45]. To select the most affected terms, we considered two factors. First, the *p-value* of the terms should be <0.05 and second, the relative number of genes which exist in each term.

### Analysis of constructed gene regulatory and TF protein-protein interaction networks

Highlighting the core component of the networks will give rise to a more comprehensive view of the expression regulation of the overall network. In this regard, apart from ontology analysis of the GRN, we applied centrality analysis to identify the most central genes of the constructed network. Degree is the simplest centrality index that calculates the number of direct neighbors of each gene which can be categorized into two indexes. The first one is out-degree, which are interactions that each gene makes with its neighbor, and the second one is in-degree which are interactions that each gene receives from other ones. We used out-degree to find the most central regulators of the GRN. In-degree and out-degree were computed for directed GRNs using the CentiScaPe App for Cytoscape [47] (Fig 1).

To predict significant protein complexes during hepatic cell identity induction from fibroblasts, we dissected our constructed TF protein-protein interaction network using the MCODE App of Cytoscape [48]. Predicted protein complexes with scores of more than two were considered to be the most significant protein complexes (Fig 1).

## Results

In the current study, we used high throughput expression data sets from six independent experiments that generated hepatocyte-like cells from mouse and human fibroblasts to draw our conclusions (Table 1). These data sets were generated from different labs using different strategies and different combinations of iHep-driving factors, including using episomal and viral vectors or using small molecules in combination with transcription factors (TFs). This analysis of different independent data sets leads to more reliable insights into the regulation of the gene expression program during the generation of iHeps from fibroblasts. Here, we identify the main affected biological processes and their regulators during direct conversion. In addition, we construct a core gene regulatory network (GRN) for the main regulators in order to understand their cross talk during the generation of hepatocyte-like cells (Fig 1).

### Regulation of the gene expression program

We used seven independent comparisons for mouse organisms to identify differentially expressed genes (DEGs) that are commonly affected during the induction of fibroblasts to a hepatocyte-like fate across all samples. To call affected genes DEGs, they should show significant expression changes in at least four of the seven comparisons. In addition, they should also have the same pattern in at least four comparisons. Based on these criteria, we found 671 DEGs during the direct conversion process, in which 247 were up-regulated and 424 were down-regulated (S1 Table). Ontology analysis of up-regulated genes clearly revealed liver-related processes and functions, including epithelial cell differentiation, organic hydroxy compound metabolic process, regulation of protein secretion, protein localization to membrane, steroid metabolic process, and alcohol metabolic process. On the other hand, for down-regulated genes, we found fibroblast specific processes ranked as the most significant processes, including regulation of locomotion, cell migration, regulation of cell motility, anatomical structure morphogenesis, regulation of cellular component movement, and extracellular matrix organization. Therefore, during the induction of iHeps from fibroblasts, common DEGs generally appear to fit into two categories; either hepatic-specific functions or fibroblast-specific functions. Furthermore, it appears that hepatic-specific processes are significantly up-regulated, whereas fibroblast-specific functions are down-regulated.

To gain more insight into the regulation of the gene expression program during the induction of fibroblasts to iHeps, we looked for TFs involved in the regulation of DEGs and in the most affected biological processes. Collectively, we found 41 differentially expressed TFs (DE-TFs) to be involved in the regulation of DEGs during the induction of hepatocyte-like fate from fibroblasts. Besides constructing this more accurate gene regulatory network for direct conversion, we also analyzed the expression of hepatic-specific factors in the ChEA database, which did not contain ChIP experiments for mice, but did contain the data for humans. To this aim, we found human ChIP experiments for these factors and analyzed the correlation of their targets in both humans and mice. Finally, we found two TFs, *Hnf4a* and *Foxa2*, for which their expressions significantly changed during direct reprogramming in both mice and humans. The expression of *Hnf4a* and *Foxa2* targets were also compared in mice and humans, and interestingly, their target genes showed a significant correlation between the two organisms. For both *Hnf4a* and *Foxa2*, 78 percent of their target genes showed full correlation between both organisms. We added *Hnf4a*, *Foxa2* and their target genes to our data in order to construct a more accurate and complete gene regulatory network. In contrast to DEGs, the expression of most DE-TFs increased, so that, 28 out of 43 TFs were up-regulated whereas 15 of 43 were down-regulated.

**Fig 2:**
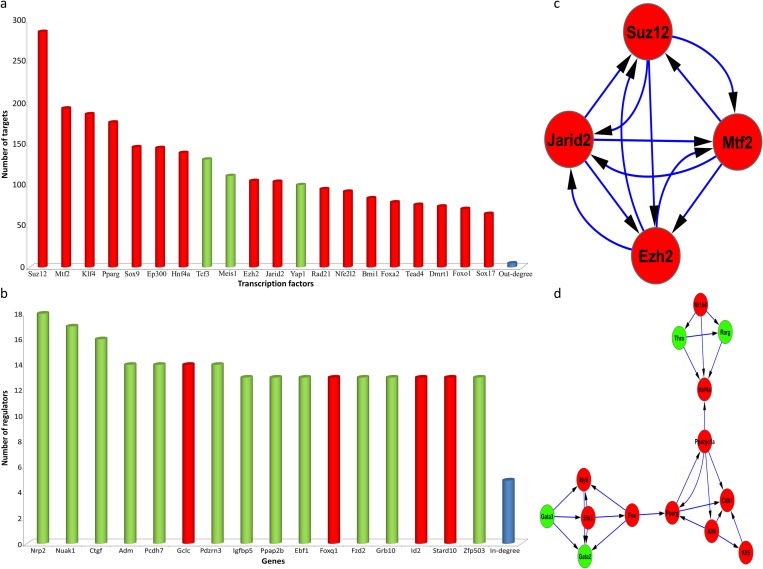
Centrality and protein complexes during induction of hepatic fate from fibroblasts. (A) Out-degree analysis was used to find the main regulators of the constructed GRN (B) and in-degree analysis was applied to identify the most regulated genes. (C & D) show significant protein complexes identified in DE-TF protein-protein interaction networks. Red and green colors show up- and down-regulation, respectively.

A gene regulatory network was constructed to obtain more data regarding the accurate role of each DE-TF in the regulation of the gene expression program. Centrality analysis using degree index was applied on the constructed network (Fig 2A). Interestingly, top regulators could be categorized into two subsets. The first subset included chromatin modifying enzymes, for example *Suz12*, *Mtf2*, *Ezh2*, and *Jarid2*, which are polycomb repressive complex2 (PRC2) members. The second subset included developmentally important factors for the generation of hepatocytes, including *Hnf4a*, *Foxa2*, *Sox17*, *Eomes*, and *Tead4*. In addition to finding the main TFs involved in regulation of gene expression, we found the most regulated genes. These genes were involved in different biological terms, including metabolic process, cellular senescence, and heart development. For example, Nuak1, a regulator of cellular senescence for which its down-regulation extends the replicative time of fibroblasts [49], was found to be heavily regulated (Fig 2B).

Furthermore, to find more details regarding the function of identified DE-TFs during hepatic fate induction, we constructed protein-protein interaction networks for DE-TFs based on validated and predicted interactions. Constructed protein-protein interaction based only on validated interactions was subjected to protein complexes analysis. Our analysis revealed a single significant protein complex which contained PRC2 members (Fig 2C). On the other hand, analysis of protein-protein interaction network constructed using both predicted and validated interactions revealed one protein complex which contained HNF4A (Fig 2D). In addition, this complex contained KLF4 and PPARG proteins, which were also seen to play roles as main regulators of DEGs (Fig 2D).

In summary, analyzing common DEGs from different data sets revealed two patterns in gene expression regulation during the generation of hepatocyte-like cells from fibroblasts. First, there is a hepatic-specific pattern in which expression of those genes are significantly up-regulated, and second a fibroblast-specific pattern in which expression of those genes are dramatically down-regulated. In addition, we found the regulators of DEGs and the biological processes in which they are involved in. Mainly, we found PRC2 and hepatocyte-specific factors, including *Hnf4a* and *Foxa2*, as main players of direct conversion.

### The role of PRC2 and hepatocyte-specific factors in the regulation of the gene expression program

**Fig 3:**
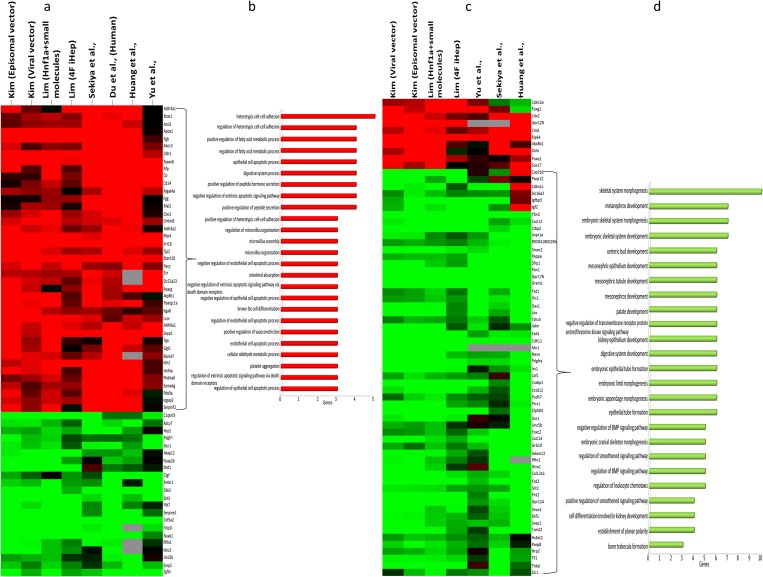
Clustering and ontology analysis of hepatic-specific TFs and PRC2 targets. (A) Clustering of common targets of Hnf4a and Foxa2 TFs and (B) ontology analysis of common targets of Hnf4a and Foxa2. Terms were selected based on the *p-value* and were ordered by number of genes in each term. (C) Clustering of common targets of PRC2 members, including Suz12, Mtf2, Ezh2, and Jarid2 and (D) ontology analysis of these common target genes. Most affected biological processes were filtered by *p-values* and then ordered by the number of DEGs. Red color shows up-regulation, whereas green color indicates down-regulation.

We found that PRC2 and hepatocyte-specific factors, including *Hnf4a* and *Foxa2*, show significant changes in the gene expression program and play dramatic roles in the regulation of gene expression during the induction of hepatic fate from fibroblasts. Here, we provide more details regarding their roles in the regulation of direct reprogramming. *Hnf4a* and *Foxa2* show 64 common targets (Fig 3A), for which 65 percent of these target genes are up-regulated. In our DEG list, only 37 percent of genes were up-regulated and 63 percent of DEGs were down-regulated.

Comparison of the targets of *Hnf4a* and *Foxa2* with the DEG list showed considerable enrichment for these two factors. Therefore, it seems that *Hnf4a* and *Foxa2* play crucial roles in setting up the hepatic gene expression program during direct conversion. In addition, up-regulated targets of *Hnf4a* and *Foxa2* were analyzed and showed significant enrichment of hepatocyte functions, specifically metabolic processes, for example in: regulation of fatty acid process, digestive system process, and cellular metabolic processes or other aspects known to be important for hepatocyte biology, like heterotypic cell-cell adhesion (Fig 3B) [50]. Collectively, these results indicate that *Hnf4a* and *Foxa2* show notable common target genes for which a large portion of these target genes are up-regulated and that these up-regulated genes have considerable roles in hepatic-specific functions.

In addition to *Hnf4a* and *Foxa2*, the role of PRC2 during the regulation of the generation of iHeps from fibroblasts was analyzed. PRC2 and its core catalytic enzyme, *Ezh2*, play significant roles during development through the silencing of gene expression by methylation of H3K27. Here, we found common target genes for all four identified members, including *Suz12*, *Mtf2*, *Ezh2* and *Jarid2*. From 68 of the common targets of PRC2 during the induction of hepatic fate, only ten of them were up-regulated and 58 of them were down-regulated (Fig 3C). Therefore, these results show that PRC2 is mainly involved in the down-regulation of the gene expression program. Ontology analysis reveals that most of the down-regulated genes are related to mesodermal development, for example: skeletal system morphogenesis, and metanephros development. Therefore, it seems that PRC2, through the down-regulation of genes involved in mesoderm development, prepare cells for their transformation into endodermal hepatocytes (Fig 3D).

Apart from *Hnf4a*, *Foxa2* and PRC2 members, we sought to identify enrichment of other main regulators, including *Klf4*, *Pparg*, *Sox9*, and *Ep300* (S2 Table). Our results showed no significant enrichment for these factors in comparison with Hnf4a, Foxa2, and PRC2 members. For example, our DEG list showed that 37 percent of genes were up-regulated and 63 percent were downregulated (S2 Table). For Sox9 target genes, 67 percent were down-regulated and 33 percent were upregulated, and showed no significant enrichment in comparison with our DEG list.

In summary, *Hnf4a* and *Foxa2* increase the expression of hepatic-specific genes whereas PRC2 members favor the direct reprogramming process though the down-regulation of genes involved in mesodermal cells development. Finally, it seems that crosstalk between hepatic-specific TFs and PRC2 members cooperatively drive the gene expression program toward the generation of iHeps from fibroblasts.

### Core regulatory network for DE-TFs and their crosstalk

**Fig 4:**
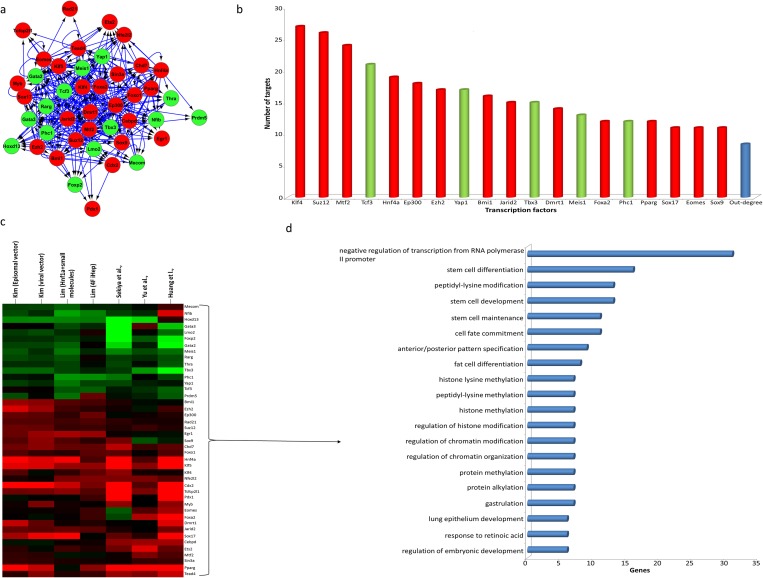
Core gene regulatory network, centrality, clustering and ontology analysis of DE-TFs. (A) Core gene regulatory network constructed for DE-TFs involved in the regulation of the gene expression program during the induction of hepatocyte-like cells and (B) the most central regulators of the core regulatory network of DE-TFs, (C) clustering analysis of DE-TFs and (D) ontology analysis of DE-TFs and the most affected biological processes of which these DE-TFs and gene regulatory network control. Most affected biological processes were filtered by *p-values* and then ordered by the number of DEGs.

Hitherto, we found 43 DE-TFs that play roles in the regulation of the gene expression program and of biological process during the induction of fibroblasts to a hepatic fate. To understand the regulatory interactions between DE-TFs and the processes in which they contribute, we constructed a core regulatory network for DE-TFs. This network was analyzed to find the main regulators of DE-TFs and the most significant and altered processes (Fig 4A). This network harbored 43 DE-TFs that were connected through 362 regulatory interactions (Fig 4A). Connectivity analysis revealed *Klf4* as the top regulator of DE-TFs during the conversion of fibroblasts into iHeps. In addition to *Klf4*, as expected, PRC2 members and hepatocyte-specific factors played significant roles in the regulation of the expression of DE-TFs (Fig 4B). Furthermore, a major component of the PRC1 complex *Bmi1* also played a considerable role in the regulation of DE-TF expression. This factor was however not found to be one of the main regulators of DEGs in the constructed gene regulatory network (Fig 2A).

We also studied the expression correlation of DE-TFs and the processes for which they contribute to during the direct conversion of fibroblasts into iHeps (Fig 4C and 4D).

Interestingly, biological process analysis showed contributions of DE-TFs in two main subsets of processes, including stem cell and developmentally related processes, and chromatin modifications involved in the regulation of gene expression. In the list of developmentally related process, we found processes which contribute to the development of endoderm and hepatocytes, including anterior/posterior pattern specification, gastrulation, and formation of primary germ layer (Fig 4D and S3 Table). For example, *Eomes*, *Ets2*, *Foxa2*, *Hnf4a*, *Klf4*, *Sox17*, and *Tcf3* were found to be involved in the gastrulation process, and all of them except *Tcf3* were found to be up-regulated. Also, *Egr1*, *Ep300*, *Foxa2*, *Hnf4a*, *Pdx1*, and *Sin3a* DE-TFs were found to be involved in metabolic processes, including response to carbohydrate, hexose, and glucose, and were also up-regulated (Fig 4D and S3 Table). On other hand, TFs that contribute to developmental processes such as lung epithelium development, fat cell development, and endocrine system development, were mostly down-regulated. For example, *Foxp2*, *Nfib*, *Thra*, *Yap1* which contribute to lung epithelium development were down-regulated, and *Gata2* and *Gata3* which play roles during the generation of fat cells and in the endocrine system were down-regulated (Fig 4C and 4D, and S3 Table). In terms of chromatin modifications that play roles in controlling gene expression, the results clearly showed the involvement of PRC2 in related terms and processes, including peptidyl-lysine modification, histone lysine methylation, and histone methylation (Fig 4D). For instance, *Ep300*, *Jarid2*, *Mtf2*, *Myb*, and *Sin3a* were involved in the positive regulation of chromosome organization. Collectively, analysis of the core regulatory network for DE-TFs showed that the correlation of hepatic-specific factors with chromatin modifying enzymes led to the generation of hepatocyte-like cells, and that these processes showed significant developmental and metabolic similarities with hepatocytes.

**Fig 5:**
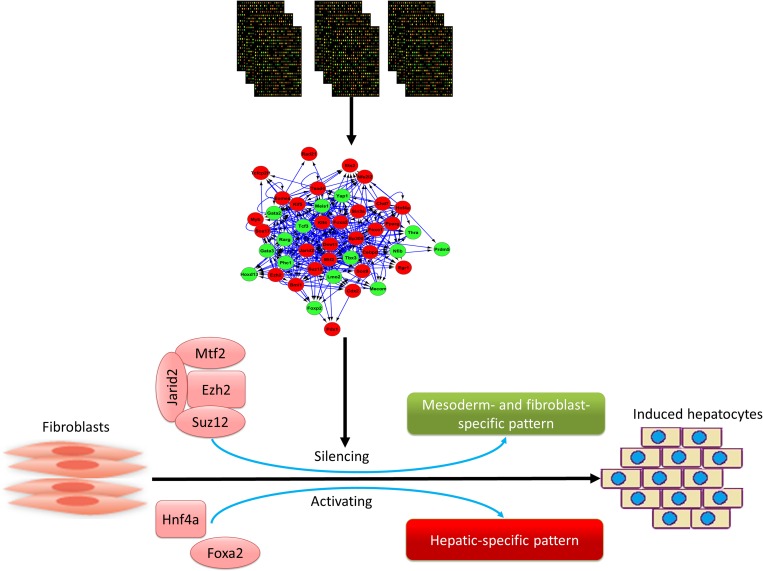
Role of hepatic-specific factors and PRC2 complex in direct conversion of fibroblasts into iHeps. Schematic view of the main results of this study, including PRC2 members which were mainly involved in the suppression of mesoderm and fibroblast specific pattern, and *Hnf4a* and *Foxa2* which were mainly involved in the upregulation of hepatic-specific pattern. Red color shows up-regulation and green shows down-regulation.

In summary, we analyzed high throughput microarray and RNA-seq data sets from different sources to find common DEGs during the induction of iHep from fibroblasts (Fig 5). These DEGs were categorized into two groups. The first group were up-regulated hepatic-specific genes which were mainly co-expressed and regulated by hepatic-specific TFs, especially *Hnf4a* and *Foxa2* (Fig 5). The second group were down-regulated genes that were related to mesoderm development and fibroblast functions and were basically controlled by PRC2 members during direct reprogramming (Fig 5). In addition, the core regulatory network of DE-TFs revealed crosstalk between these factors and their roles in regulating each other and developmental process. Also, hepatic-specific factors and PRC2 members were found to be the main players of the core-regulatory network of DE-TFs. Collectively, our results show that the generation of iHeps from fibroblast during direct reprogramming progresses occurs through the co-operation of hepatic factors, especially *Hnf4a* and *Foxa2* and PRC2 members including *Suz12*, *Mtf2*, *Ezh2*, and *Jarid2*. Hepatic-specific factors lead to the up-regulation of genes involved in the generation and function of hepatocytes, whereas PRC2 members favor the direct conversion process through the down-regulation of mesoderm and fibroblast-specific genes.

## Discussion

In the current study, we dissected the gene expression profiles during the direct conversion of fibroblasts into iHeps in order to understand the molecular mechanisms underlying the regulation of the gene expression program. Collectively, we found that direct conversion of fibroblasts into iHeps are governed in parallel by hepatic-inducing factors and factors which take part in silencing fibroblast-specific genes. In the following parts, we will discuss the role of these factors in direct reprogramming and also clarify the similarity of their functions during normal development or differentiation of hepatocytes from pluripotent stem cells.

We found that *Hnf4a* and *Foxa2* TFs regulate the expression of a significant number of common DEGs across all samples, and also show the same expression pattern in mice and humans. In addition, we found these factors to be the main regulators of DEGs in the GRN and of DE-TFs in the core regulatory network. Despite the fact that Hnf4a and Foxa2 have been used in many studies for the generation of hepatocyte-like cells from fibroblasts [5,6,26–29], their exact roles in regulating the gene expression program are still not very well understood. In contrast to direct reprogramming, the roles of these factors in the regulation of gene expression program and cell fate decision have been studied comprehensively during development and differentiation.

Previously, the FoxA family of TFs was identified to be involved in the regulation of hepatic specification and differentiation. During gastrulation, Foxa2 was found to be expressed in the anterior primitive streak, which subsequently generates definitive endoderm and where Foxa2 expression remains high [51]. In addition to the anterior primitive streak and the definitive endoderm, this factor is expressed in the notochord and the mesoderm during development [52,53]. Our results confirmed the importance of Foxa2 in these processes (Fig 4D). In addition, polarity and proper cellular junctions are abolished in endodermal cells that carry the mutant Foxa2 gene [54]. Foxa2 is also involved in the establishment of epithelial fate while also inhibiting mesenchymal fate [54]. Studies show that in mouse embryo, deficiency for both FoxA1 and FoxA2 severely abolished the expression of the Afp gene, which in turn diminished the capacity of the endoderm to respond properly to extracellular signals and subsequently induced hepatic specification [55,56]. Foxa1 and Foxa2 are known as pioneer transcription factor during the specification of hepatocyte because of their ability to displace the repressive histone linker H1 and expose DNA to other TFs to initiate the expression program toward a hepatocyte fate [57,58].

A significant and central role of hepatocyte nuclear factor 4 (HNF4A) had been found to be in the regulation of all functional characteristics of human hepatocytes [59], which we also obtained through our analysis (Fig 3B). In humans, HNF4A clearly plays a role in the hepatic specification of definitive endoderm cells during differentiation. Using shRNA, DeLaForest et al (2011) diminished HNF4A expression, which subsequently disrupted many functions related to hepatocytes, including lipid metabolism, small molecule biochemistry, carbohydrate metabolism, and molecular transport [60]. Interestingly, these processes were also identified in our analyses (Fig 3B and S3 Table). Furthermore, HNFA4 was previously found to play a role in the regulation of amino acid metabolism, cholesterol metabolism, and steroid metabolism [61], which we also saw in our analysis (Fig 3B and S3 Table). Importantly during the differentiation of pluripotent stem cells toward hepatocyte-like cells, we found HNF4A to be directly involved in the regulation of cell junction-related genes (Fig 3B). HNF4A has previously been shown to regulate the expression of genes related to all kinds of cell junctions and adhesion, including tight junctions, adherence junctions, desmosomes, and gap junctions, through its binding to 29 promoters of 16 genes during the differentiation of PSCs into hepatocyte like cells [61]. A previous study has revealed the role HNF4A in stabilizing the complex network of TFs in hepatocyte cells [62], a fact also seen in our results (Fig 4A and 4B). In addition, ChIP-chip studies in adult liver cells revealed that this factor was involved in the regulation of its own promoter in addition to other main liver transcription factors, which maintain the functionality and stability of liver [63]. Lastly, HNF4A has been shown to play a central role in the gene network that regulates the expression of hepatocyte TFs in the embryonic liver [61].

The above studies regarding the roles of *Hnf4a* and *Foxa2* in both development and the generation of functional hepatocytes are extremely similar with the processes regulated by these factors during the induction hepatic fate from fibroblasts. Therefore, it seems that *Hnf4a* and *Foxa2* mimic developmental processes during the generation of iHeps from fibroblasts.

In addition to Hnf4a and Foxa2, a number of studies used additional or completely different sets of hepatic-specific TFs including Hnf1a, Hnf1b, Foxa1, and Foxa3 [5,6,29]. We analyzed the expression of these TFs in our data and found that they were not significantly differentially expressed in at least three independent studies, and thus not categorized as DE-TFs and removed from our analysis. *Hnf1a*, *Hnf1b*, and *Foxa3* have been identified as DEGs in data sets from studies by Huang et al. (2011), and Sekiya et al. (2011) [5,6]. We used common DEGs and DE-TFs with defined criteria for our analysis, so despite the fact that these factors have been used in a number of studies, the heterogeneity in their expression precluded their use in our analysis.

Although there is a lack of studies regarding the role of PRC2 members in the direct generation of iHeps from fibroblasts, their importance during development and during the reprogramming of somatic cells into pluripotent state are better understood. More recently, the role of PRC2 during development, during differentiation toward a hepatic fate, and during its maturation into a hepatocyte were documented. PRC2 has a very significant role in maintaining the pluripotent characteristics of pluripotent stem cells and also in preparing them for correct differentiation upon receiving the appropriate signals [64]. Ezh2, via its methyl transferase activity, acts as a core catalytic member of the PRC2 and has a very important role in normal development. It was reported that mouse embryos deficient for Ezh2 cannot properly complete the early stages of development [65]. In addition, it was shown that Suz12 is crucial for the precise development of mice. The absence of Suz12, is concomitant with the misregulation of genes which are important for mouse development [66]. Suz12 mainly occupies the promotor regions of TFs and of developmentally determinant genes in embryonic stem cells, and helps regulate their expression [67]. The functionality of PRC2 depends on the collaboration of Ezh2 and Suz12 with other members For example, it has been demonstrated that embryonic stem cells (ESCs) deficient for Eed also have dramatically compromised levels of Ezh2 protein [68].

Emerging evidences show a crucial contribution of PRC2 members in the regulation of the gene expression program during different stages of hepatocyte generation. For example, it was shown that in ESCs with Suz12 knockdown are unable to generate endoderm cells [69]. Also, it was documented that ESCs with Ezh2 and Eed deficiency show dramatic abnormalities in their differentiation and commitment toward mesoendodermal cells [69]. After specification of hepatic cells from a pancreatic lineage, Ezh2 is a crucial regulator of the growth and maturation of specified hepatocytes [70,71]. This specification from a pancreatic lineage occurs through histone acetylation by Ep300 factor followed by histone methylation by Ezh2 [70,71]. It was further shown that loss of Ezh2 leads to considerably lower amounts of liver tissue at E8.5 [70,71]. More interestingly, the role of Ezh2 was shown in later stages of hepatocyte development and proliferation, where the self-renewal characteristics of hepatocyte progenitor cells were abolished upon deprivation of Ezh2 [72]. Loss of Ezh2 activity caused changes in levels of H3K27me3, which abolished hepatic progenitor maturation and liver maturation, revealing a crucial role for this factor [73]. Furthermore, a recent study revealed the important roles of EZH1 and EZH2 in the normal physiology of the liver. Bae et al., (2015) showed that deficiency of these two factors led to the low regeneration capacity of liver cells during injury and toxic stress [74].

In recent years, many reports have demonstrated the role of PRC2 members in reprogramming, besides their roles in development. For example, it was shown that the Ezh2 core catalytic of PRC2, which is also a H3K27 methyl-transferase, plays a crucial role during the reprogramming of somatic cells into pluripotency states [75–77]. It has been shown that the expression of Ezh2 continually increased during the reprogramming of fibroblasts into a pluripotency state [78,79]. In addition, it has been well-documented that not only did repressing expression of this methyl-transferase lead to a significant reduction of reprogramming efficiency [76,77], but also that overexpressing it could enhance the efficiency and numbers of the obtained iPSC clones [77,79]. Experiments on other members of the PRC2 complex, Eed, Jarid2, Mtf2, and Suz12, showed that down-regulation of these factors resulted in decreased reprogramming efficiency [76,80]. Jarid2 and Mtf2 function as modulators of PRC2, and also act as repressors of developmentally important genes during the conversion of somatic cells into iPSCs [80]

Taken together, our results show significant roles for PRC2 during the induction of hepatic fate from fibroblasts. Our systems biology approach revealed that PRC2 was involved in the silencing of mesoderm- and fibroblast-specific gene expression. Also, PRC2 members present in the same protein complex (Fig 2C) were found to be the main regulators of DEGs and DE-TFs. In addition, it seems that PRC2 in cooperation with *Ep300* repress pancreatic lineage-specific gene expression and favor the generation of hepatocytes.

In addition to *Hnf4a*, *Foxa2*, and the PRC2 complex, we also identified other TFs that could play a role during direct reprogramming. For example, using Yamanaka factors including Oct4/Pou5f1, Sox2, Klf4, and Myc in combination with lineage specific factors has been another strategy for direct reprogramming process [81]. The significant presence of *Klf4* in the regulation of DEGs and DE-TFs (Fig 2A and Fig 4A and 4B) supports the fact that *Klf4* can be used as a candidate factor to increase the efficiency of the induction of hepatic fate from fibroblasts. For example, interestingly, Lim et al. (2016) showed that using Klf4 and Myc in combination with hepatic-specific factors led to a significant increase in the efficiency of the generation of iHeps from fibroblasts [28]. Also, this study showed Klf4 and Myc are not crucial for the induction hepatocyte-like cells but increase the efficiency of the process. Therefore, it seems that testing other identified master regulators in combination with hepatic-specific factors or PRC2 members could lead to better protocols for the generation of iHeps from fibroblasts.

It will also be useful to consider other possible interactions and roles for the DE-TFs we identified. For example, it was previously shown that HNF4A and TCF4 recognize considerable numbers of common DNA sequences and compete together to regulate the expression of these shared genes [82]. It is possible that this may be occurring for *Hnf4a* and *Tcf3* TFs, and since we found the hepatic-specific factor *Hnf4a* to be up-regulated and *Tcf3* to be down-regulated, the direct reprogramming process might be favored (Fig 4B and 4C). In addition, some TFs that may play roles during direct conversion may not have been identified using our analysis for several reasons, including heterogeneity in expression, lack of high throughput data or lack of related information in sources and databases. For example, we found *Pparg* to be a DE-TF that plays a role in the regulation of the gene expression program, but we did not find *Ppara* as a regulator of the constructed gene regulatory network. Because of the importance of *Ppara* in the metabolism of liver cells [83], it also might play a role during the induction to a hepatic fate from fibroblasts. *Ppara* was considered a DE-TF in five out of the eight comparisons in our analysis (data not shown), but the ChEA database from which we recruited TF-binding site data sets did not include any data sets for *Ppara*, therefore we did not consider it in our subsequent analysis.

In conclusion, our results revealed significant details regarding the factors and mechanisms involved in the regulation of the gene expression program during the generation of iHeps from fibroblasts. The eight independent comparisons used in our study provide a comprehensive view on the regulation of gene expression. Interestingly, we found two distinct groups of biological processes that were mainly controlled by two groups of regulators. Up-regulated DEGs were mainly categorized as hepatic-specific genes, and were co-expressed and regulated by hepatic-specific TFs, especially *Hnf4a* and *Foxa2*. On other hand, down-regulated genes were categorized as mesoderm- and fibroblast-specific genes, and their expression levels were mainly repressed by PRC2 members, including *Suz12*, *Mtf2*, *Ezh2*, and *Jarid2*. Altogether, using systems biology approaches, we clarified the role of *Hnf4a* and *Foxa2* as hepatic-specific TFs, and for the first time, we introduced the PRC2 complex as the main regulator that favors the direct reprogramming process in cooperation with hepatic-specific factors.

## Supporting Information

S1 Table List of common DEGs during induction to cardiomyocyte-like cells from mouse fibroblasts. (XLSX)

S2 Table Target enrichement analysis for Klf4, Pparg, Sox9, and Ep300 DE-TFs. (DOCX)

S3 Table Ontology results for DE-TFs core regulatory network. (XLSX)
