# Supplementary material for: Role of Hepatic-Specific Transcription Factors and Polycomb Repressive Complex 2 during Induction of Fibroblasts to Hepatic Fate
Source: PLoS One. 2016 Nov 30;11(11):e0167081. doi: 10.1371/journal.pone.0167081 (PMC5130264; doi:10.1371/journal.pone.0167081)
Supplement: S2 Table — (DOCX) [file pone.0167081.s002.docx]

| TFs | Nr of targets | Up-regulated | Down-regulated | Percent of up^*^ | Percent of down^*^ |
| --- | --- | --- | --- | --- | --- |
| Klf4 | 186 | 69 | 117 | 0.37 | 0.63 |
| Pparg | 182 | 61 | 121 | 0.335 | 0.665 |
| Sox9 | 146 | 48 | 98 | 0.33 | 0.67 |
| Ep300 | 145 | 42 | 103 | 0.29 | 0.71 |

Table S2. Enrichment analysis of Klf4, Pparg, Sox9, and Ep300 (Up-regulated TFs)

^*^In our DEG list, only 37 percent of genes were up-regulated and 63 percent of genes were down-regulated.
